# Interaction of immune cells with renal cancer development: Mendelian randomization (MR) study

**DOI:** 10.1186/s12885-024-12196-8

**Published:** 2024-04-09

**Authors:** Zhongwen Lu, Yu Yin, Tian Rao, Xinchi Xu, Kai Zhao, Zhanpeng Liu, Chao Qin, Min Tang

**Affiliations:** The State Key Lab of Reproductive, Department of Urology, The First Affiliated Hospital of Nanjing Medical University, Nanjing, Jiangsu 210009 China

**Keywords:** RCC, Mendelian randomization, Immune cells, Tumor microenvironment

## Abstract

**Background:**

Renal cell carcinoma (RCC) is a prevalent and extensively immune-infiltrated malignancy of the urinary system. Immune cells play a crucial role in both the progression and therapeutic interventions targeting RCC. Nevertheless, the interplay between RCC and immune cells remains understudied, lacking substantial evidence supporting their causal relationship.

**Methods:**

For the purpose of investigating the causal connection between RCC and immune cell characteristics, a two-way two-sample Mendelian randomization (MR) analysis was carried out in this study. The aim was to determine whether specific immune cell traits have a causal impact on the risk of RCC. In order to achieve this, publicly accessible genetic data was utilized to examine and establish the potential relationship between 731 immune cell characteristics and the likelihood of developing RCC. Additionally, various techniques were applied to verify the reliability, variability, and presence of horizontal pleiotropy in the outcomes.

**Results:**

We found a bidirectional causal relationship between RCC and immune cells according to the MR analysis results. It should be noted that CD4-CD8-T cells (OR = 1.61, 95%CI = 1.02–2.55, *P* = 4.07 × 10^–2^) pose a risk for RCC, whereas BAFF-R (OR = 0.69, 95%CI = 0.53–0.89, *P* = 5.74 × 10^–3^) and CD19 (OR = 0.59, 95%CI = 1.02–2.55, *P* = 4.07 × 10^–2^) on B cells act as protective factors. Furthermore, the presence of RCC reduces the levels of B cells (OR = 1.05, 95%CI = 1.01–1.09, *P* = 1.19 × 10^–2^) and CD8 + T cells (OR = 1.04, 95%CI = 1.00–1.08, *P* = 2.83 × 10^–2^).

**Conclusions:**

Our research illustrates the intricate correlation between immune cells and RCC, presenting novel insights for the prospective safeguarding against RCC risk and the exploration of fresh therapeutic targets.

**Supplementary Information:**

The online version contains supplementary material available at 10.1186/s12885-024-12196-8.

## Introduction

As the prevailing histologic subtype of kidney cancer, renal cell carcinoma (RCC) accounts for approximately 80 to 85% of all primary renal neoplasms [1]. The incidence of RCC has been steadily rising by 2% annually worldwide over the last twenty years [2, 3]. It is estimated that there will be around 81,000 newly diagnosed cases and nearly 15,000 deaths of RCC in the United States in 2023 [4]. Currently, a variety of effective therapies, for example: surgical management and adjuvant therapy, are available for patients with RCC. Surgery, such as radical nephrectomy and partial nephrectomy, is the mainstay for curative treatment of RCC, but the surgical trauma and postoperative complication may lead to distress in patients. Moreover, a considerable proportion of patients with locally advanced tumors in RCC are susceptible to experiencing recurrence. So, despite emerging diagnostic and therapeutic strategies, the understanding of etiology and new molecular therapeutic targets remains urgently to be expanded [5].

The immune microenvironment has been widely recognized as a significant factor in both the development and regulation of RCC. Immune cells, various cytokines and tissue factors play an important role in tumorigenesis and immune escape. It has been demonstrated that RCC is highly infiltrated by T cells [6–9]. The presence of Th17 cells and a higher CD8^+^ T/Treg ratio have been found to be correlated with enhanced survival rates in clear cell renal cell carcinoma(ccRCC) patients, while the presence of Th2 cells and Tregs have been associated with unfavorable outcomes [9]. Cytokines and inflammatory factors are produced at the site of infection by activated helper immune cells, leading to endocrine changes that affect body function [10, 11]. IL-15 can inhibit the progression of RCC by influencing ILC1 to induce innate anti-tumor immune responses [10]. These results suggest that immunologic approaches and targeted molecular agents represent an important strategy. Nowadays, studies on the association between tumor microenvironment and RCC mainly focus on T cells, and little attention has been paid to the association between other immune cells, cytokines and RCC.

Mendelian randomization (MR) is an analytical method that uses single nucleotide polymorphism (SNP) as an instrumental variable (IV) to infer the causal relationship between two traits, which can minimize the influence of confounding factors [12–15]. In this study, we performed a comprehensive two-sample bidirectional MR analysis to determine the causal relationship between immune cell traits and RCC. We found a bidirectional causal relationship between immune cells and RCC, with both influencing each other, revealing a complex link between RCC and immune cells.

## Materials and methods

### Study design

**Fig. 1:**
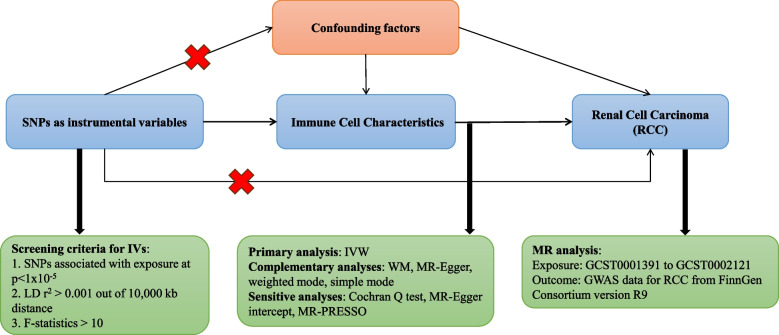
Flowchart of the entire Mendelian Randomization analysis

Based on two-sample MR Analysis, we evaluated the causal relationship between immune cell characteristics and RCC. Instrumental variables (IVs) in MR Analysis must satisfy three key assumptions: (1) IVs is closely related to exposure; (2) IVs was not associated with possible confounding factors; (3) IVs affects the outcome only through exposure and not by other pathways [16]. The populations we studied in this study were all European and American. The population study was approved by the relevant institutional review boards and the subjects provided informed consent.An overview of the analytical approach is shown in Fig. 1.

### Data sources

GWAS summary statistics for each immune cell are publicly available from the GWAS Catalog (accession numbers from GCST0001391 to GCST0002121) [12, 17]. A total of 731 immune characteristic phenotypes were included in the data, which were divided into 7 immune cells according to classification, namely NK cells, myeloid cells, B cells, monocytes, Treg cells, cDC cells and mature T cells. RCC related GWAS data can be got from ieu [18]. The immunocyte-related GWAS data utilized in this study are based on a cohort of 6,602 participants of European descent from the central eastern coast of Sardinia, Italy. The RCC (Renal Cell Carcinoma)-related GWAS data employed in our analysis were sourced from IEU, comprising a total of 174,006 European ancestry samples, with a ncase count of 971.

### Selection of instrumental variables (IVs)

In order to select suitable IVs, we followed the following criteria: (1) SNPs associated with genome-wide motif significance threshold (*P* < 5 × 10^–8^) were selected as candidate IVs. Unfortunately, only a very small fraction of SNPs was enrolled at that threshold, and we therefore used a second threshold (1 × 10^–5^) to screen the IVs for more comprehensive results. For the screening of IVs in RCC, since the desired SNPs could not be screened at 5 × 10^–8^, we relaxed the criteria to 1 × 10^–5^; (2) SNPS with low linkage disbalance (LD) and *R*^2^ < 0.001(aggregation window size = 10,000 kb) were selected using the sample data of the European 1000 Genomes Project as the reference panel, and only SNPS with the lowest *P*-value were retained as candidate IVs; (3) Delete palindromic SNPS; (4) The F statistic of each SNP was calculated to evaluate the strength of IVs, and SNPS with F < 10 were excluded [19]. Detailed information on SNPs can be found in Supplementary Table 1.

### Statistical analysis

In this study, anti-variance weighting (IVW), simple model, MR Egger regression, weighted median and weighted modeling were used to explore the potential causal relationship between immune cells and RCC. In addition, we use MR-PRESSO analysis to identify and mitigate horizontal pleiotropy by eliminating significant outliers. Heterogeneity of individual SNP effects was assessed using the Cochran Q test [20]. The estimates are expressed as odds ratios (ORs) with 95% confidence intervals (ci), which indicate the average change in outcomes resulting from each exposure. In this study, IVW method was used as the main analysis method, and other methods were used as auxiliary proof [21]. These results only provide evidence of a causal relationship between exposure and outcomes, with no other explanation.

The analysis was mainly carried out using statistical software R. The R packages used in MR Analysis include various software packages such as TwoSampleMR, mendelanrandomization, MRPRESSO and ggplot2 for data processing and graphic painting. *P* < 0.05 was considered statistically significant.

## Results

### Exploration of the causal effect of immune cells on RCC

**Fig. 2:**
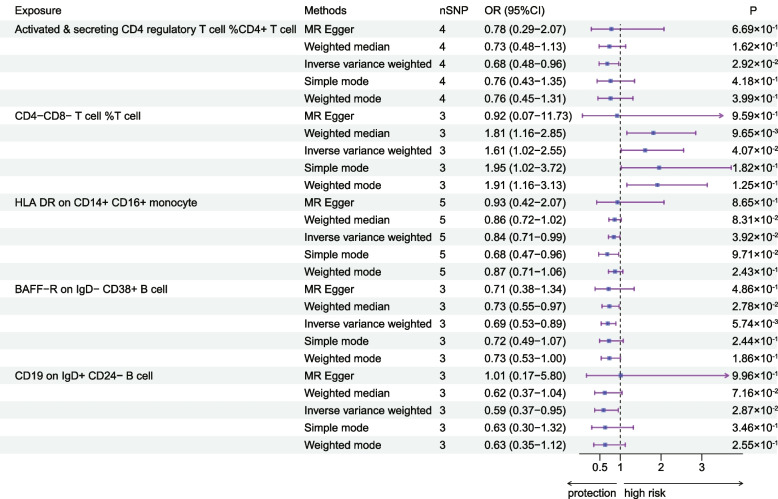
The forest plot shows the causal relationship between immune cell characteristics and RCC by different methods

To investigate the causal effect of immunophenotype on RCC, we used two-sample MR analysis with IVW method as the main analysis method. We detected that most immunophenotypes showed a protective trend against RCC: the OR for Activated & secreting CD4 regulatory T cells was 0.68 (95%CI = 0.48–0.96, *P* = 2.92 × 10^–2^), and the OR for HLADR on CD14 + CD16 + monocyte was 0.84 (95%CI = 0.71–0.99, *P* = 3.92 × 10^–2^), BAFF-R on IgD- CD38 + B cell OR of 0.69 (95%CI = 0.53–0.89, *P* = 5.74 × 10^–3^), and the same trend was observed in the weighted median approach with an OR of 0.73 (95%CI = 0.55–0.97, *P* = 2.78 × 10^–2^) and an OR of 0.59 (95%CI = 0.37–0.95, *P* = 2.87 × 10^–2^) for CD19 on IgD + CD24- B cells, in addition to which we found that the protective effect of CD19 was also reflected in other types of B cells. In contrast, CD4-CD8-T cell was a risk factor for RCC, with an OR value of 0.84 (95%CI = 0.71–0.99, *P* = 3.92 × 10^–2^), and the fact that CD4 + CD8 + T cell presented a protective effect against RCC also proved the above point (Fig. 2, Table 1, Supplementary Table 2). Heterogeneity and horizontal pleiotropy among the screened IVs were excluded by MR-Egger with the test of pleiotropy (Supplementary Table 3), and the stability of the results was also demonstrated by scatterplots and funnel plots (Supplementary Fig. 1, Supplementary Fig. 2). MR analysis at another threshold also confirmed the reliability of our conclusions (Supplementary Table 4). MR analysis of the UKBB database as well as the FinnGen database also demonstrated the relevance of our conclusions (Supplementary Table 5, Supplementary Table 6).

**Table 1:** MR analysis of the causal relationship between immune cells and renal cancer

Exposure	Methods	nSNP	OR (95%CI)	*P*
Activated & secreting CD4 regulatory T cell %CD4 + T cell	MR Egger	4	0.78 (0.29–2.07)	6.69 × 10^–1^
	Weighted median	4	0.73 (0.48–1.13)	1.62 × 10^–1^
	Inverse variance weighted	4	0.68 (0.48–0.96)	2.92 × 10^–2^
	Simple mode	4	0.76 (0.43–1.35)	4.18 × 10^–1^
	Weighted mode	4	0.76 (0.45–1.31)	3.99 × 10^–1^
CD4-CD8- T cell %T cell	MR Egger	3	0.92 (0.07–11.73)	9.59 × 10^–1^
	Weighted median	3	1.81 (1.16–2.85)	9.65 × 10^–3^
	Inverse variance weighted	3	1.61 (1.02–2.55)	4.07 × 10^–2^
	Simple mode	3	1.95 (1.02–3.72)	1.82 × 10^–1^
	Weighted mode	3	1.91 (1.16–3.13)	1.25 × 10^–1^
HLA DR on CD14 + CD16 + monocyte	MR Egger	5	0.93 (0.42–2.07)	8.65 × 10^–1^
	Weighted median	5	0.86 (0.72–1.02)	8.31 × 10^–2^
	Inverse variance weighted	5	0.84 (0.71–0.99)	3.92 × 10^–2^
	Simple mode	5	0.68 (0.47–0.96)	9.71 × 10^–2^
	Weighted mode	5	0.87 (0.71–1.06)	2.43 × 10^–1^
BAFF-R on IgD- CD38 + B cell	MR Egger	3	0.71 (0.38–1.34)	4.86 × 10^–1^
	Weighted median	3	0.73 (0.55–0.97)	2.78 × 10^–2^
	Inverse variance weighted	3	0.69 (0.53–0.89)	5.74 × 10^–3^
	Simple mode	3	0.72 (0.49–1.07)	2.44 × 10^–1^
	Weighted mode	3	0.73 (0.53–1.00)	1.86 × 10^–1^
CD19 on IgD + CD24- B cell	MR Egger	3	1.01 (0.17 − 5.80)	9.96 × 10^–1^
	Weighted median	3	0.62 (0.37 − 1.04)	7.16 × 10^–2^
	Inverse variance weighted	3	0.59 (0.37 − 0.95)	2.87 × 10^–2^
	Simple mode	3	0.63 (0.30 − 1.32)	3.46 × 10^–1^
	Weighted mode	3	0.63 (0.35 − 1.12)	2.55 × 10^–1^

### Exploration of the causal effect of RCC on immune cells

**Fig. 3:**
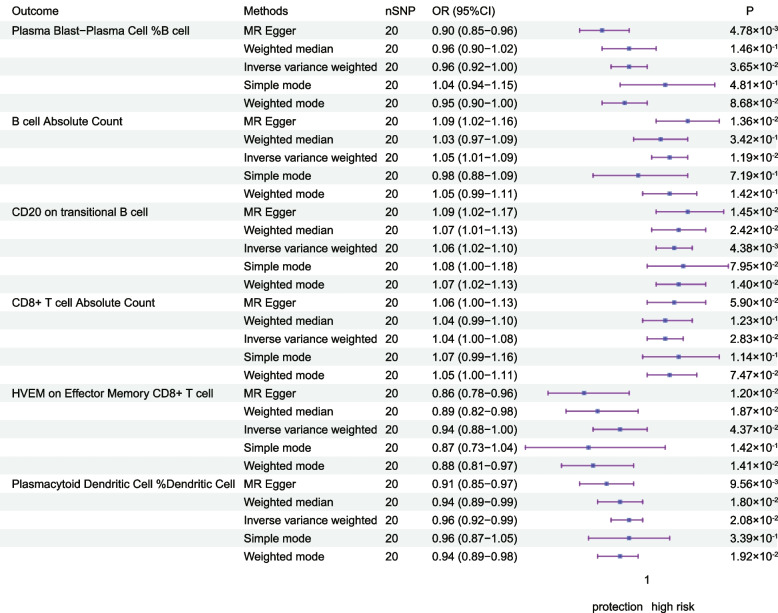
The forest plot shows the causal relationship between RCC and immune cell characteristics by different methods

To explore the causal effect of RCC on immune cells, we performed an inverse Mendelian randomization analysis, and we identified that the affected immune cells were mainly concentrated in T cells versus B cells. We found that with the onset of RCC can Plasma Blast-Plasma Cell expression levels (OR = 0.96, 95%CI = 0.92–1.00, *P* = 3.65 × 10^–2^), and the same trend was observed in the MR-Egger method (OR = 0.90, 95%CI = 0.85–0.96, *P* = 4.78 × 10^–3^).The expression level of HVEM on Effector Memory CD8 + T cell was also found to be increased (OR = 0.94, 95%CI = 0.88–1.00, *P* = 4.37 × 10^–2^). As for B cell,we found that RCC was negatively correlated with the expression level of B cell, and the same trend was observed by IVW method (OR = 1.05, 95%CI = 1.01–1.09, *P* = 1.19 × 10^–2^) and MR Egger (OR = 1.09, 95%CI = 1.02–1.16, *P* = 1.36 × 10^–2^) method. Meanwhile, the estimation of the occurrence of RCC for CD8 + T cells was 1.04 (95%CI = 1.00–1.08, *P* = 2.83 × 10^–2^), with the same trend, although the other methods were not statistically significant (Table 2, Fig. 3).Effector Memory CD4 + T cells (OR = 1.04, 95% CI = 1.00–1.08, *P* = 4.51 × 10–2) and Effector Memory CD8 + T cell (OR = 1.04, 95%CI = 1.00–1.08, *P* = 4.51 × 10^–2^) also observed a similar trend (Supplementary Table 7). And we excluded the presence of horizontal pleiotropy and heterogeneity by MR-Egger's intercept, MR-PRESSO's test, and heterogeneity test (Supplementary Table 8). Scatterplots and funnel plots also indicate the stability of the results (Supplementary Fig. 3, Supplementary Fig. 4).

**Table 2:** MR analysis of the causal relationship between renal cancer and immune cells

Outcome	Methods	nSNP	OR (95%CI)	*P*
Plasma Blast-Plasma Cell %B cell	MR Egger	20	0.90 (0.85–0.96)	4.78 × 10^–3^
	Weighted median	20	0.96 (0.90–1.02)	1.46 × 10^–1^
	Inverse variance weighted	20	0.96 (0.92–1.00)	3.65 × 10^–2^
	Simple mode	20	1.04 (0.94–1.15)	4.81 × 10^–1^
	Weighted mode	20	0.95 (0.90–1.00)	8.68 × 10^–2^
B cell Absolute Count	MR Egger	20	1.09 (1.02–1.16)	1.36 × 10^–2^
	Weighted median	20	1.03 (0.97–1.09)	3.42 × 10^–1^
	Inverse variance weighted	20	1.05 (1.01–1.09)	1.19 × 10^–2^
	Simple mode	20	0.98 (0.88–1.09)	7.19 × 10^–1^
	Weighted mode	20	1.05 (0.99–1.11)	1.42 × 10^–1^
CD20 on transitional B cell	MR Egger	20	1.09 (1.02–1.17)	1.45 × 10^–2^
	Weighted median	20	1.07 (1.01–1.13)	2.42 × 10^–2^
	Inverse variance weighted	20	1.06 (1.02–1.10)	4.38 × 10^–3^
	Simple mode	20	1.08 (1.00–1.18)	7.95 × 10^–2^
	Weighted mode	20	1.07 (1.02–1.13)	1.40 × 10^–2^
CD8 + T cell Absolute Count	MR Egger	20	1.06 (1.00–1.13)	5.90 × 10^–2^
	Weighted median	20	1.04 (0.99–1.10)	1.23 × 10^–1^
	Inverse variance weighted	20	1.04 (1.00–1.08)	2.83 × 10^–2^
	Simple mode	20	1.07 (0.99–1.16)	1.14 × 10^–1^
	Weighted mode	20	1.05 (1.00–1.11)	7.47 × 10^–2^
HVEM on Effector Memory CD8 + T cell	MR Egger	20	0.86 (0.78–0.96)	1.20 × 10^–2^
	Weighted median	20	0.89 (0.82–0.98)	1.87 × 10^–2^
	Inverse variance weighted	20	0.94 (0.88–1.00)	4.37 × 10^–2^
	Simple mode	20	0.87 (0.73–1.04)	1.42 × 10^–1^
	Weighted mode	20	0.88 (0.81–0.97)	1.41 × 10^–2^
Plasmacytoid Dendritic Cell %Dendritic Cell	MR Egger	20	0.91 (0.85–0.97)	9.56 × 10^–3^
	Weighted median	20	0.94 (0.89–0.99)	1.80 × 10^–2^
	Inverse variance weighted	20	0.96 (0.92–0.99)	2.08 × 10^–2^
	Simple mode	20	0.96 (0.87–1.05)	3.39 × 10^–1^
	Weighted mode	20	0.94 (0.89–0.98)	1.92 × 10^–2^

## Discussion

Based on publicly available GWAS data, we categorized 731 immune cell traits into 7 categories and explored the causal relationship between them and RCC. We found that there are few MR analysis to explore the causal relationship between immune traits and RCC. In this study, we found a causal relationship between immune phenotypes such as T-cells, B-cells, and monocytes and the risk of developing RCC, as well as influencing the expression levels of some immune cells as RCC develops.

Our research revealed a positive correlation between the incidence of RCC and the elevated levels of CD4-CD8-T cells, whereas CD4 + CD8 + T cells would be the ones to show a protective effect. CD4-CD8-T cells, a type of regulatory T cells (Tregs), do not express NK cells markers. The function of regulatory T cells (Tregs) is mainly in maintaining immune cell homeostasis while suppressing anti-tumor immune responses [22, 23]. Research has unequivocally demonstrated that CD4 + T cells possess a cytotoxic regimen that effectively eliminates malignant cells. Moreover, the functional capabilities of CD4 + T cells primarily revolve around the generation of cytokines, whereas the ability to directly eradicate target cells resides within the CD8 + T cell subset. Consequently, elevated quantities of CD4 + CD8 + T cells hold the potential to impede the development and advancement of tumors. This aligns perfectly with the outcomes of our investigation. Additionally, scientific evidence supports the utilization of CD4/8 cells as an immunotherapeutic strategy for renal cell carcinoma (RCC) [24, 25]. Previous research has demonstrated that the survival rate of patients with RCC can be enhanced by having a higher proportion of tumor-infiltrating NK cells and Th1 markers (such as T-cells expressing HLA-DR +) [26]. Our study further reveals that there is a protective effect of HLA DR on the incidence of RCC by affecting the CD14 + CD16 + monocyte panel. HLA-DR is a cell surface receptor belonging to the MHC class II, which is encoded by the human leukocyte antigen complex on chromosome 6 region 6P21. In cases of chronic inflammation, the reduced expression of HLA-DR in monocytes confirms the anti-inflammatory role of this molecule and further supports the validity of our findings [12, 27]. Our study suggests the possibility that HLA-DR could be a therapeutic target for RCC, other investigations have also illustrated a correlation between RCC and HLA ligands, suggesting that specific HLA-presenting peptides unique to ccRCC might serve as potential targets for immunotherapy [28–30].

BAFF-R is among the trio of receptors detected on fully developed B-cells, exhibiting abilities to proficiently eradicate diverse B-cell malignancies. Simultaneously, following genetic reconfiguration to foster the expression of CD19-specific chimeric antigen receptor (CAR), it can be effectively utilized for addressing progressive B-cell neoplasms, boasting robust antineoplastic outcomes. Currently, several investigations have illustrated the potential of CD19/BAFF-R as a novel therapeutic target for cancer [31–34], aligning with our own research findings that CD19 and BAFF-R on B-cells bestow protective properties. Bevacizumab, an extensively used anticancer medication, has been found to primarily target CD19, an essential gene-enriched pathway. Furthermore, it affectes T-cell impairment, particularly affecting CD19 [35, 36]. CD19's impact on the effectiveness of sunitinib, another antitumor drug, has also been observed [37]. These findings highlight the potential of our discovery to offer innovative therapeutic targets for treating RCC.

This study explored the bidirectional causal analysis between immune cells and RCC through a two-sample Mendelian randomization study, which was statistically efficient because of the large sample size of the study population in this experiment. In addition, we used multiple MR analysis methods while excluding the effects of confounding factors, so our results are robust. Nowadays, immune-related studies on RCC are mainly focused on T cells, and our study can focus the immune studies on RCC among other immune cells and suggest new ideas and causal correlations. However, the study has some limitations. First, MR analysis can only reveal the causal association between the two, and molecular experiments are needed to confirm the mechanism by which immune cells influence the occurrence of RCC. Second, this is an overall data and lacks individual information to further stratify the population. Third, as to the mechanisms, it may not be caused by just one immune phenotype, but rather multiple immune phenotypes acting together, hence the discrepancy that arises when studying a single factor. Finally, we relaxed the screening criteria for IVs of RCC, and these may generate some false positives.

## Conclusions

In conclusion, in this study we demonstrated a causal association between immune cells and RCC through bidirectional MR analysis, highlighting the complex pattern of interactions and interactions between the immune system and RCC. In addition, this study provides ideas for exploring the biological mechanisms between RCC and immune cells, and the found immune cells may become key molecules for early intervention in RCC development and treatment of RCC.

### Supplementary Information

**Supplementary Material 1.**

**Supplementary Material 2.**

**Supplementary Material 3.**

**Supplementary Material 4.**

**Supplementary Material 5.**

**Supplementary Material 6.**

**Supplementary Material 7.**

**Supplementary Material 8.**

**Supplementary Material 9.**

## Data Availability

Data is provided within the manuscript or supplementary information files. GWAS summary statistics for each immune cell are publicly available from the GWAS Catalog (accession numbers from GCST0001391 to GCST0002121) and RCC related GWAS data can be got from IEU (https://gwas.mrcieu.ac.uk/) and FinnGen Consortium version R9 (https://r9.finngen.fi/).
