# Supplementary material for: Interaction of immune cells with renal cancer development: Mendelian randomization (MR) study
Source: BMC Cancer. 2024 Apr 9;24:439. doi: 10.1186/s12885-024-12196-8 (PMC11005164; doi:10.1186/s12885-024-12196-8)
Supplement: Supplementary file 1 — Supplementary Material 1. [file 12885_2024_12196_MOESM1_ESM.docx]

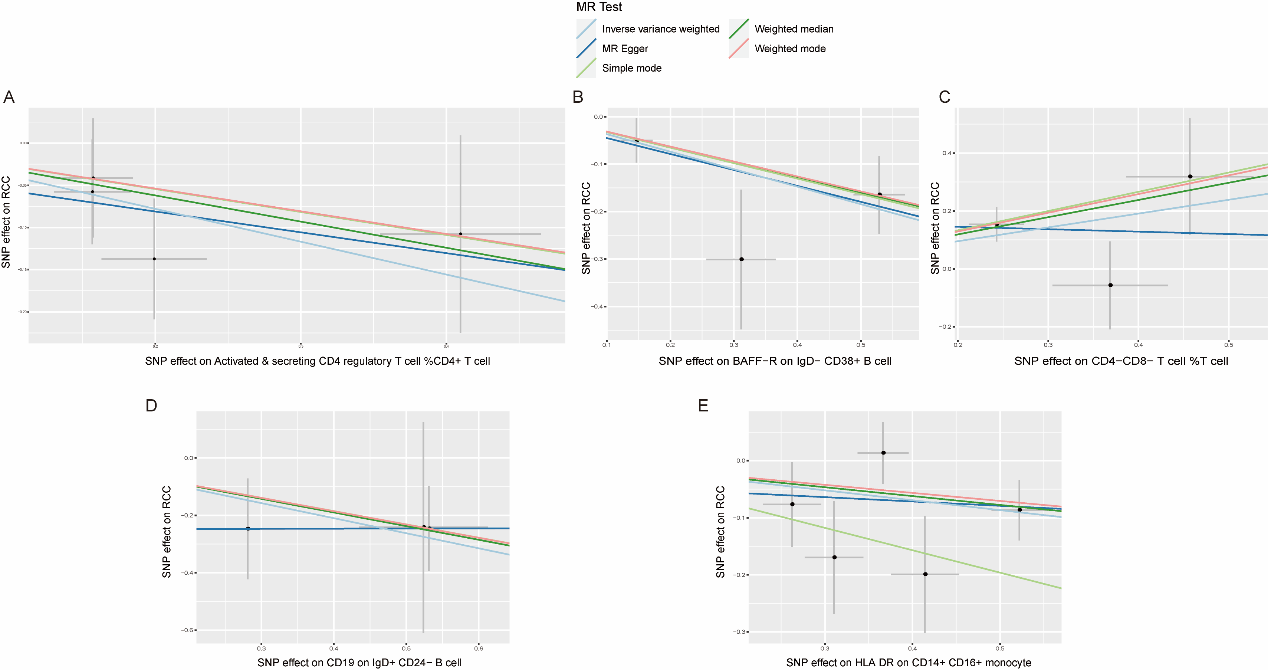


**Supplementary Figure 1.** Scatter plots between immune cells and RCC.


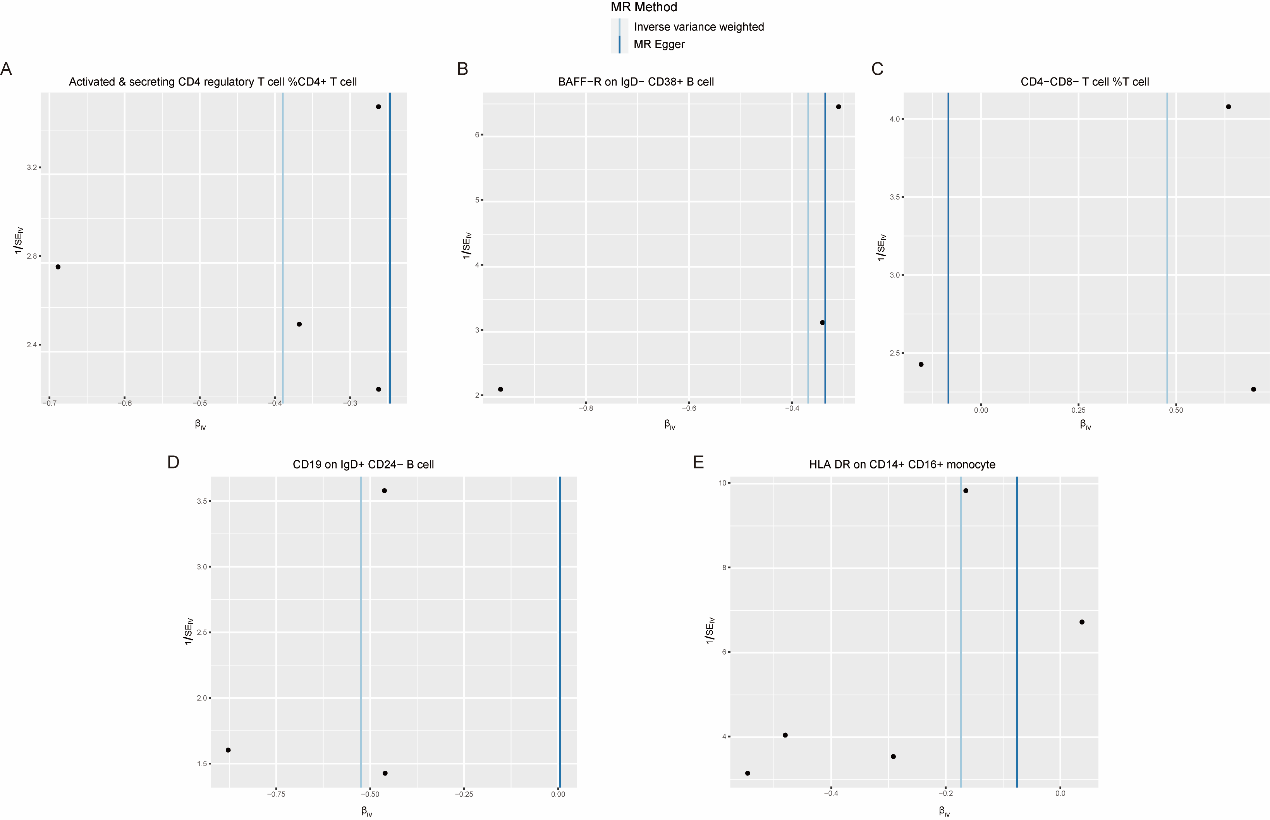


**Supplementary Figure 2.** Funnel plots between immune cells and RCC.


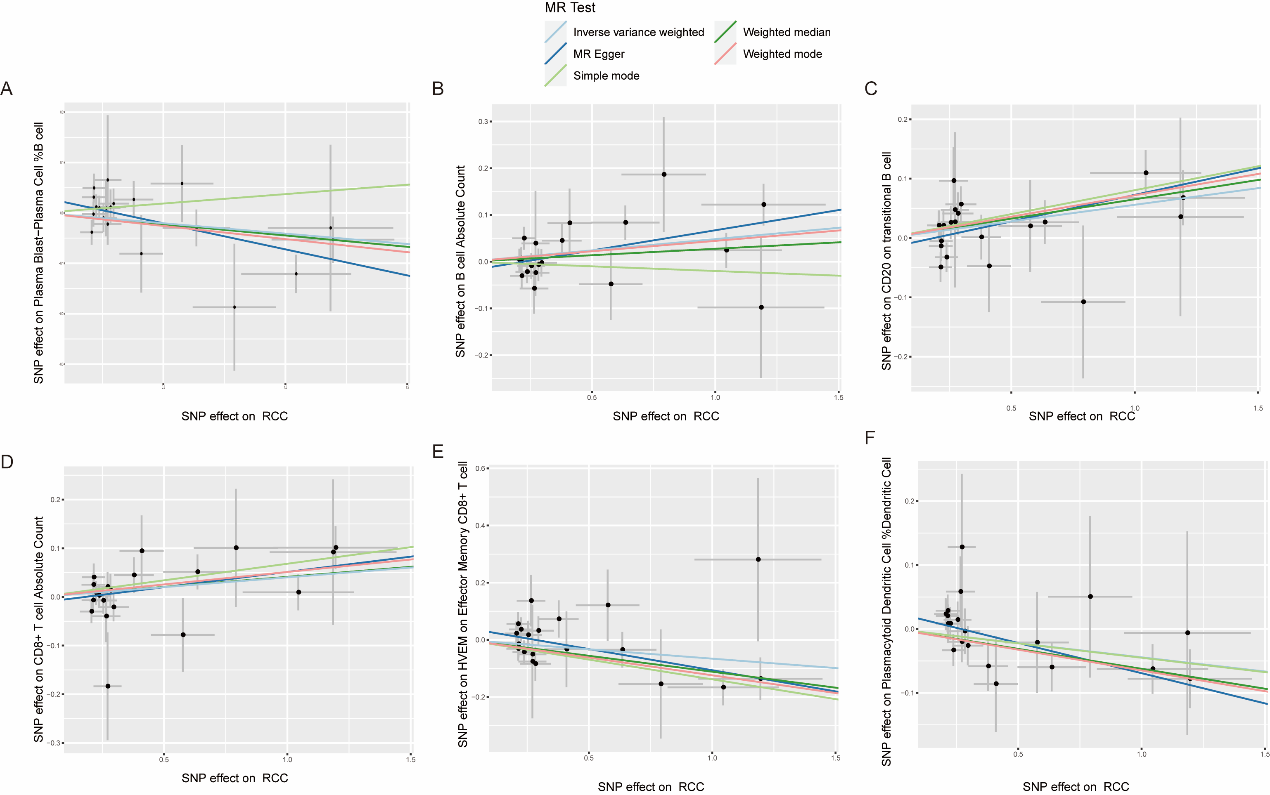


**Supplementary Figure 3.** Scatter plots between RCC and immune cells.


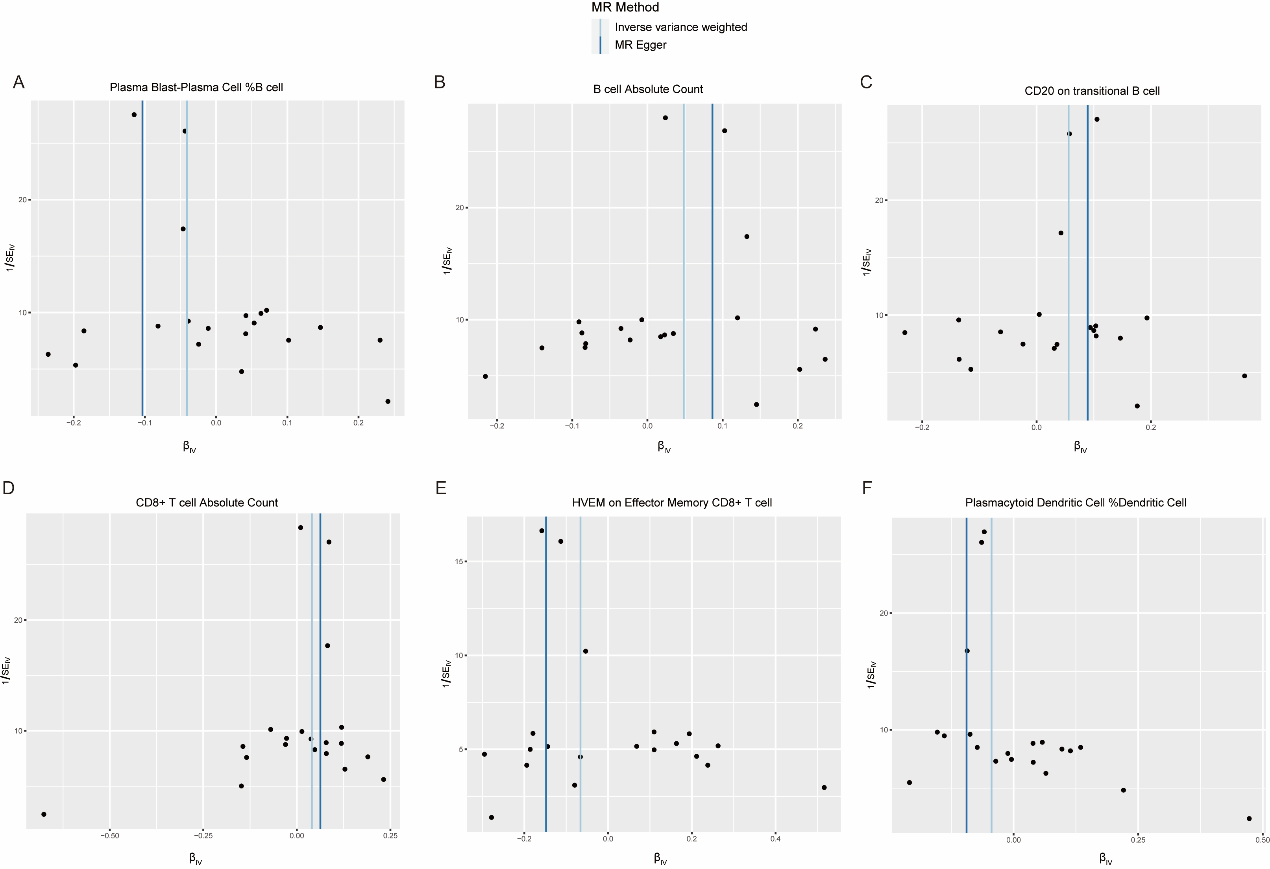


**Supplementary Figure 4.** Funnel plots between RCC and immune cells.
